# What Type of Prostate Cancer Is Systematically Overlooked by Multiparametric Magnetic Resonance Imaging? An Analysis from the PROMIS Cohort

**DOI:** 10.1016/j.eururo.2020.04.029

**Published:** 2020-08

**Authors:** Joseph M. Norris, Lina M. Carmona Echeverria, Simon R.J. Bott, Louise C. Brown, Nick Burns-Cox, Tim Dudderidge, Ahmed El-Shater Bosaily, Eleni Frangou, Alex Freeman, Maneesh Ghei, Alastair Henderson, Richard G. Hindley, Richard S. Kaplan, Alex Kirkham, Robert Oldroyd, Chris Parker, Raj Persad, Shonit Punwani, Derek J. Rosario, Iqbal S. Shergill, Vasilis Stavrinides, Mathias Winkler, Hayley C. Whitaker, Hashim U. Ahmed, Mark Emberton

**Affiliations:** a UCL Division of Surgery & Interventional Science, University College London, London, UK; b London Deanery of Urology, London, UK; c Department of Urology, University College London Hospitals NHS Foundation Trust, London, UK; d Department of Urology, Frimley Health NHS Foundation Trust, London, UK; e Medical Research Council (MRC) Clinical Trials Unit, University College London, London, UK; f Department of Urology, Taunton & Somerset NHS Foundation Trust, Taunton, UK; g Department of Urology, University Hospital Southampton NHS Foundation Trust, Southampton, UK; h Department of Radiology, Royal Free London NHS Foundation Trust, London, UK; i Department of Pathology, University College London Hospitals NHS Foundation Trust, London, UK; j Department of Urology, Whittington Health NHS Trust, London, UK; k Department of Urology, Maidstone & Tunbridge Wells NHS Trust, Tunbridge Wells, UK; l Department of Urology, Hampshire Hospitals NHS Foundation Trust, Hampshire, UK; m Department of Radiology, University College London Hospitals NHS Foundation Trust, London, UK; n Public & Patient Representative, Nottingham, UK; o Department of Academic Urology, The Royal Marsden NHS Foundation Trust, Sutton, UK; p Department of Urology, North Bristol NHS Trust, Bristol, UK; q Department of Urology, Sheffield Teaching Hospitals NHS Foundation Trust, Sheffield, UK; r Department of Urology, Wrexham Maelor Hospital NHS Trust, Wrexham, UK; s Department of Urology, Imperial College Healthcare NHS Trust, London, UK; t Imperial Prostate, Division of Surgery, Department of Surgery & Cancer, Faculty of Medicine, Imperial College London, London, UK

**Keywords:** False negative magnetic resonance imaging, Undetected cancer, Multiparametric magnetic resonance imaging, PROMIS, Prostate cancer

## Abstract

**Background:**

All risk stratification strategies in cancer overlook a spectrum of disease. The Prostate MR Imaging Study (PROMIS) provides a unique opportunity to explore cancers that are overlooked by multiparametric magnetic resonance imaging (mpMRI).

**Objective:**

To summarise attributes of cancers that are systematically overlooked by mpMRI.

**Design, setting, and participants:**

PROMIS tested performance of mpMRI and transrectal ultrasonography (TRUS)-guided biopsy, using 5 mm template mapping (TPM) biopsy as the reference standard.

**Outcome measurements and statistical analysis:**

Outcomes were overall and maximum Gleason scores, maximum cancer core length (MCCL), and prostate-specific antigen density (PSAD). Cancer attributes were compared between cancers that were overlooked and those that were detected.

**Results and limitations:**

Of men with cancer, 7% (17/230; 95% confidence interval [CI] 4.4–12%) had significant disease overlooked by mpMRI according to definition 1 (Gleason ≥ 4 + 3 of any length or MCCL ≥ 6 mm of any grade) and 13% (44/331; 95% CI 9.8–17%) according to definition 2 (Gleason ≥ 3 + 4 of any length or MCCL ≥ 4 mm). In comparison, TRUS-guided biopsy overlooked 52% (119/230; 95% CI 45–58%) of significant disease by definition 1 and 40% (132/331; 95% CI 35–45%) by definition 2. Prostate cancers undetected by mpMRI had significantly lower overall and maximum Gleason scores (*p* = 0.0007; *p* < 0.0001) and shorter MCCL (median difference: 3 mm [5 vs 8 mm], *p* < 0.0001; 95% CI 1–3) than cancers that were detected. No tumours with overall Gleason score > 3 + 4 (Gleason Grade Groups 3–5; 95% CI 0–6.4%) or maximum Gleason score > 4 + 3 (Gleason Grade Groups 4–5; 95% CI 0–8.0%) on TPM biopsy were undetected by mpMRI. Application of a PSAD threshold of 0.15 reduced the proportion of men with undetected cancer to 5% (12/230; 95% CI 2.7–8.9%) for definition 1 and 9% (30/331; 95% CI 6.2–13%) for definition 2. Application of a PSAD threshold of 0.10 reduced the proportion of men with undetected disease to 3% (6/230; 95% CI 1.0–5.6%) for definition 1 cancer and to 3% (11/331; 95% CI 1.7–5.9%) for definition 2 cancer. Limitations were post hoc analysis and uncertain significance of undetected lesions.

**Conclusions:**

Overall, a small proportion of cancers are overlooked by mpMRI, with estimates ranging from 4.4% (lower boundary of 95% CI for definition 1) to 17% (upper boundary of 95% CI for definition 2). Prostate cancers undetected by mpMRI are of lower grade and shorter length than cancers that are detected.

**Patient summary:**

Prostate cancers that are undetected by magnetic resonance imaging (MRI) are smaller and less aggressive than those that are detected, and none of the most aggressive cancers are overlooked by MRI.

## Take Home Message

Few prostate cancers are undetected by multiparametric magnetic resonance imaging (mpMRI), and these are lower in size and grade, than prostate cancer that is detected by mpMRI. This reinforces the role that mpMRI should play in the assessment of suspected prostate cancer.

## 1 Introduction

The introduction of multiparametric magnetic resonance imaging (mpMRI) has enhanced risk stratification for men at risk of prostate cancer, beyond the traditional standard of serum prostate-specific antigen (PSA) and systematic transrectal ultrasound (TRUS)-guided prostate biopsy [1], [2], [3], [4]. It is now generally accepted that mpMRI has the greatest validity and reliability among all our diagnostic methods. Its role in the diagnostic process is now considered a central one [5], [6], [7], [8], [9].

However, it is also acknowledged that mpMRI does not detect all prostate cancers. Some have argued that this is one of the most valuable attributes [9]. Microfocal Gleason 3 + 3 (generally perceived as indolent disease) can often be overlooked [10]. Indeed, mpMRI detection is positively associated with grade, volume, and stage [11], [12], [13]. The larger and more aggressive the cancer, the greater the probability of detection [14], [15], [16], [17]. However, there are concerns that a number of potentially clinically significant tumours can be overlooked by mpMRI. The literature demonstrates a wide variation in proportions of overlooked cancer, ranging between 7% and 55% [1], [11] depending on study methodology and definitions of significant disease.

The Prostate MR Imaging Study (PROMIS) was a multicentre, paired-cohort, confirmatory study that compared the diagnostic performance of mpMRI versus traditional systematic TRUS-guided biopsy against the most stringent reference standard possible. Each of the 576 men included in the final PROMIS analysis underwent prebiopsy mpMRI, followed by systematic TRUS-guided biopsy and concurrent transperineal template mapping (TPM) biopsy (the reference test) in which biopsies were taken at 5 mm intervals across the entire prostate. The analyses presented in this paper report in detail the attributes of cancers (defined by a priori definitions 1 and 2) that were detected by mpMRI at 1.5 T, compared with cancers that were overlooked.

## 2 Patients and methods

### 2.1 Study population

**Fig. 1:**
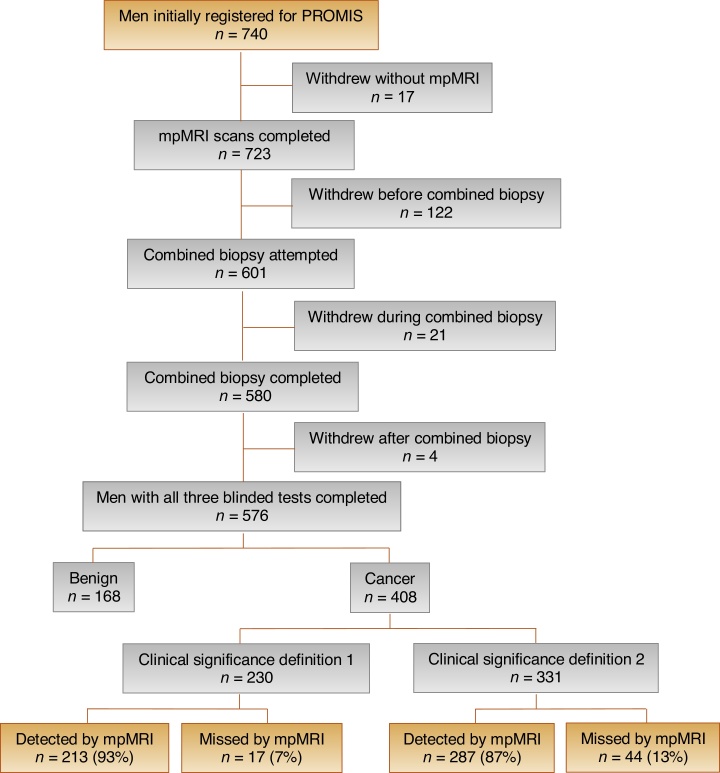
Flow chart for study inclusion. *n* = sample size; mpMRI = multiparametric magnetic resonance imaging; MRI = magnetic resonance; PROMIS = Prostate Magnetic Resonance Imaging Study.

In brief, PROMIS was a multicentre study in which biopsy-naïve men with PSA ≤ 15 ng/mL underwent prebiopsy 1.5 T mpMRI followed by a combined biopsy procedure under general anaesthesia. The mpMRI parameters used are reported in full in the main PROMIS report [1]. Combined biopsy consisted of standard systematic TRUS biopsy along with simultaneous 5 mm transperineal TPM biopsy. TRUS-guided biopsy was carried out after TPM. Each test was performed and reported blinded to results. PROMIS was registered on ClinicalTrials.gov (NCT01292291). The study protocol for PROMIS has been described in depth elsewhere [1], [18]. For the present study, all men who met the definition of clinically significant disease (by either definition) were identified for analysis (Fig. 1). Ethical approval for PROMIS was granted by the National Research Ethics Service Committee London (Ref: 11/LO/0185).

### 2.2 Definitions of clinical significance

Clinically significant prostate cancer was defined using the two definitions outlined in PROMIS [1]. Definition 1 for clinically significant disease was overall Gleason score ≥ 4 + 3 of any length or maximum cancer core length (MCCL) ≥ 6 mm of any grade. Definition 2 for clinically significant disease was overall Gleason score ≥ 3 + 4 of any length or MCCL ≥ 4 mm of any grade. These criteria were developed and validated for TPM biopsy for the detection of Gleason score ≥ 4 [19] and cancer core lengths representative of lesions ≥ 0.5 mL [20], [21], [22], [23].

### 2.3 Post hoc analysis

Once stratified by each definition of clinical significance, men were divided into mpMRI-detected (Likert score 3–5) and mpMRI-undetected (Likert score 1–2) groups. An additional threshold of tumour visibility was also evaluated (mpMRI-detected group: Likert score 4–5; mpMRI-undetected group: Likert score 1–3). Outcome measures for this post hoc analysis were based upon data gathered during PROMIS, including overall Gleason score per patient, maximum Gleason score per needle, MCCL per patient, and PSA density (PSAD). PSAD was calculated by dividing serum PSA by mpMRI-derived prostate volume (using the prolate ellipsoid method). Overall Gleason score was defined as the predominant Gleason pattern across the entire prostate and constituted the final pathological score. The maximum Gleason score was defined as the highest Gleason pattern found in any biopsy core.

### 2.4 Statistical analysis

We described the characteristics for each with mpMRI-detected and mpMRI-undetected cancer, and then stratified analysis according to two definitions of clinical significance. Mean values with standard deviations and median values with interquartile ranges were calculated with descriptive statistical techniques to characterise the measures of central tendency for demographic patient data, MCCL measurements, and PSAD values. All outcome data were unpaired and had non-normal distribution, and as such, two-sided nonparametric statistical tests were used. Overall and maximum Gleason scores were compared with the chi-square test, and MCCL and PSAD values were compared with the Mann–Whitney U test. Alpha level was 0.05 for all statistical tests. All analyses were conducted using GraphPad Prism 8 (Graph-Pad Software, Inc., La Jolla, CA, USA) and the R statistical environment.

## 3 Results

### 3.1 Overall detection

Demographic patient data for all 576 men included in the final PROMIS analysis are shown in Table 1. We identified that significant prostate cancer was not detected by mpMRI in 7% (17/230; 95% confidence interval [CI] 4.4–12%) of men according to definition 1 and in 13% (44/331; 95% CI 9.8–17%) of men according to definition 2 (Supplementary Fig.1; Supplementary Table 1). The addition of systematic TRUS-guided biopsy would have missed 59% (10/17; 95% CI 33–82%) of definition 1 cancers undetected by mpMRI and 70% (31/44; 95% CI 55–83%) of definition 2 cancers undetected by mpMRI (Supplementary Table 2).

**Table 1:** Summary of demographic data for all patients within PROMIS.

Characteristic	
Sample size, *n*	576
Age (yr), mean (SD)	63.4 (7.6)
PSA (ng/mL), mean (SD)	7.1 (2.1)
BMI (kg/m^2^), mean (SD)	27.8 (4.4)
Family history of PCa, *n* (%)	127 (22)
Ethnicity, *n* (%)	
White	502 (87)
Black	39 (7)
Asian	16 (7)
Mixed	6 (1)
Other	12 (2)
Overall Gleason score	
3 + 3	100
3 + 4	252
3 + 5	1
4 + 3	44
4 + 5	7
5 + 4	4
Maximum cancer core length (mm)	
1–5	186
6–10	160
11–15	59
16–20	3

BMI = body mass index; *n* = number; PCa = prostate cancer; PROMIS = Prostate Magnetic Resonance Imaging Study; PSA = prostate-specific antigen; SD = standard deviation; TPM = template mapping.

Pathological results are from TPM biopsy.

### 3.2 Cancer grade

Table 2 compares key pathological outcomes between mpMRI-detected and mpMRI-undetected prostate cancer. Significant prostate cancer undetected by mpMRI was significantly lower in overall and maximum Gleason grades than significant cancer that was detected by mpMRI (*p* = 0.0007 and *p* < 0.0001, respectively). On a per-patient basis, no overall Gleason score > 3 + 4 (Gleason Grade Groups 3–5) on TPM biopsy was undetected by mpMRI throughout the entire cohort (95% CI 0–6.4%; Table 3). On a per-needle basis, no maximum Gleason score > 4 + 3 (Gleason Groups 4–5) on TPM biopsy was undetected by mpMRI throughout the entire cohort (95% CI 0–8.0%). No overall Gleason pattern 5 (either primary or secondary) was undetected by mpMRI (95% CI 0–27%).

**Table 2:** Comparison of key histopathological outcomes of MRI-detected and MRI-undetected prostate cancer in PROMIS, by both definitions of clinical significance.

Characteristic	MRI-detected PCa (def 1)	MRI-undetected PCa (def 1)	Difference, *p* value	MRI-detected PCa (def 2)	MRI-undetected PCa (def 2)	Difference, *p* value
Sample size*, n* (%)	213 (93) (95% CI 88–96%)	17 (7) (95% CI 4.4–12%)	–	287 (86) (95% CI 83–90%)	44 (13) (95% CI 9.8–17%)	–
Overall Gleason			*p* *=* 0.0023			*p* = 0.0007
3 + 3	4.2% (9/213)	5.9% (1/17)	1.7% (95% CI –8.4% to 12%)	5.9% (17/287)	14% (6/44)	8.1% (95% CI 0.02–16%)
3 + 4	69% (148/213)	94% (16/17)	25% (95% CI 2.6–47%)	75% (214/287)	86% (38/44)	11% (95% CI 2.5–24%)
3 + 5	0.47% (1/213)	0% (0/17)	–	0.35% (1/287)	0% (0/44)	–
4 + 3	2.1% (44/213)	0% (0/17)	–	15% (44/287)	0% (0/44)	–
4 + 5	1.9% (4/213)	0% (0/17)	–	2.4% (7/287)	0% (0/44)	–
5 + 4	3.3% (7/213)	0% (0/17)	–	1.4% (4/287)	0% (0/44)	–
Overall MCCL (mm)			*p* *=* 0.14			*p* < 0.0001
1–5	3.8% (8/213)	0% (0/17)	–	29% (82/287)	61% (27/44)	32% (95% CI 17–47%)
6–10	69% (147/213)	76% (13/17)	7% (95% CI –16% to 30%)	51% (147/287)	30% (13/44)	21% (95% CI 5.1–37%)
11–15	26% (55/213)	24% (4/17)	2% (95% CI –24% to 20%)	19% (55/287)	9.1% (4/44)	9.9% (95% CI 2.2–22%)
16–20	1.4% (3/213)	0% (0/17)	–	1.0% (3/287)	0% (0/44)	–
Median (IQR)	9 (7–11)	8 (6–11)	1 (95% CI 0–2)	8 (5–10)	5 (4–6)	3 (95% CI 1–3)

CI = confidence interval; def = definition of clinical significance; IQR = interquartile range; MCCL = maximum cancer core length; MRI = magnetic resonance imaging; *n* = number; PCa = prostate cancer; PROMIS = Prostate Magnetic Resonance Imaging Study; TPM = template mapping.

Pathological results are from TPM biopsy.

**Table 3:** Proportions of prostate cancer detected and undetected by mpMRI in PROMIS, according to Gleason grade group.

GGG	MRI-detected PCa	MRI-undetected PCa	Difference
Group 1	5.9% (17/287)	14% (6/44)	8.1% (95% CI 0.02–16%)
Group 2	75% (214/287)	86% (38/44)	11% (95% CI –2.5% to 24%)
Group 3	15% (44/287)	0% (0/44)	–
Group 4	0.35% (1/287)	0% (0/44)	–
Group 5	3.8% (11/287)	0% (0/44)	–

CI = confidence interval; GGG = Gleason Ggrade Group; mpMRI = multiparametric MRI; MRI = magnetic resonance imaging; PCa = prostate cancer; PROMIS = Prostate Magnetic Resonance Imaging Study; TPM = template mapping.

Pathological results are from TPM biopsy.

### 3.3 Cancer core length

Clinically significant prostate cancer undetected by mpMRI had significantly shorter MCCL than significant cancer that was detected by mpMRI (median difference: 3 mm [5 vs 8 mm], *p* < 0.0001; 95% CI 1–3).

### 3.4 PSA density

PSAD was significantly lower for men with mpMRI-invisible disease (Supplementary Fig. 2) than for men with mpMRI-visible disease (median difference: 0.08 [0.12 vs 0.20], *p* < 0.0001; 95% CI 0.05–0.11). Application of a PSAD threshold (above which a biopsy would be indicated) altered the rates of undetected significant prostate cancer. Using a PSAD threshold of 0.15 in the context of negative mpMRI (Likert score 1–2) lowered the proportion of men with undetected disease to 5% (12/230; 95% CI 2.7–8.9%) for definition 1 cancer and to 9% (30/331; 95% CI 6.2–13%) for definition 2 cancer. Application of a PSAD threshold of 0.10 to negative mpMRI lowered the proportion of men with undetected disease to 3% (6/230; 95% CI 1.0–5.6%) for definition 1 cancer and to 3% (11/331; 95% CI 1.7–5.9%) for definition 2 cancer.

### 3.5 Alternative tumour visibility threshold

When the definition of mpMRI-undetected disease was raised to Likert 1–3, the proportion of clinically significant prostate cancers that were overlooked by mpMRI was 22% (51/230; 95% CI 17–28%) according to definition 1 and 34% (113/331; 95% CI 29–40%) according to definition 2. Overall and maximum Gleason grades were still significantly lower (*p* < 0.0001 and *p* < 0.0001, respectively), and MCCL was still significantly smaller (median difference: 4 mm [8 vs 9 mm], *p* < 0.0001; 95% CI 2–4), even with a wider definition for nondetection.

## 4 Discussion

In summary, we have shown in this post hoc analysis of the PROMIS dataset that the proportion of important cancers that are systematically overlooked by 1.5 T mpMRI is low (7%). In the least stringent setting (ie, upper limit of 95% CI for definition 2 disease detection), the estimate for clinically significant prostate cancer overlooked by mpMRI could be as a high as 17%. However, in this same situation, the upper estimate for significant cancer overlooked by systematic TRUS-guided biopsy would be 45% [1]. In contrast, in the most stringent setting (ie, lower limit of 95% CI for definition 1 disease detection), the estimate for clinically significant prostate cancer overlooked by mpMRI could be as low as 4.4%, thus highlighting the key importance of both statistical estimates and definitions of clinical significance.

Overall, our findings support the observations made by others that cancers that are overlooked by mpMRI are significantly smaller and less aggressive than those that are detected [11], [12], [13], [24]. Through evaluation of PROMIS, our analysis provides uniquely robust characterisation of significant prostate cancers that mpMRI does not detect, by using 5 mm TPM biopsy as the reference standard. This methodological strength avoids inherent biases of radical prostatectomy–correlated studies, including the following: population and selection biases; registration challenges; ex vivo tissue with 10% shrinkage, distortion, and inconsistent 5–10 mm sampling frame; and tissue loss from the trim of material to achieve full face. Aside PROMIS, there are a small number of other trials that have used saturation TPM biopsy to evaluate mpMRI accuracy. Whilst they offer advantage over radical prostatectomy–based interrogation, they remain limited by common drawbacks that PROMIS did not suffer, including retrospective single-centre design, heterogeneous uncontrolled patient populations, variable and simplistic definitions for clinical significance, and lack of evaluation of the performance of systematic TRUS-guided biopsy [25], [26], [27].

One potential limitation of our study is the reliance upon a per-patient approach, in which a single overall score was assigned to each mpMRI scan (Likert scores 1–5). The use of per-patient analysis has the benefit of mirroring a real-life diagnostic setting; however, it potentially limits detailed analysis of tumour conspicuity, as there is a possibility that men with concurrent visible and invisible tumours may have their mpMRI-invisible cancer overlooked due to an overall positive mpMRI score generated by the visible lesion. Furthermore, the addition of targeted biopsy to the PROMIS protocol would have enabled increased confidence in radiological-pathological alignment.

An additional limitation of the PROMIS dataset is that radiologists were aware of PSAD at the time of reporting, and as such, may have attributed positive mpMRI scores in cases of high PSAD, again limiting analyses of mpMRI-invisible lesions. This is important, as a recent systematic review with meta-analysis demonstrated that PSAD was the strongest predictor for clinically significant prostate cancer in the context of negative prebiopsy mpMRI [28]. An associated limitation of using PSAD thresholds to stratify men with negative mpMRI is that, in a real-world setting, men with high PSAD and negative mpMRI would be unlikely to be offered a TPM biopsy, but rather a systematic TRUS-guided biopsy, which may still overlook significant cancer in this setting.

Where our findings differ from other estimates may be explained by issues of population characteristics, mpMRI quality, study design, and definitions of risk thresholds. There are methodological issues associated with all these types of studies. Within PROMIS, we managed to avoid many of them (work-up, incorporation, and spectrum biases) as this was the rationale for the design that we chose. The fact that all components of the study (mpMRI, TRUS-guided biopsy, and TPM biopsy) were independent and blinded to each other would suggest that our estimates are as valid as they can be. The multicentre design means that different levels of expertise and competence in all three components of the study are represented. The choice of using 1.5 T was due to the fact that many studies prior to PROMIS had reported high-accuracy metrics with this magnetic field strength, and this was the norm in the UK at the time of the study; this of course means that the performance of mpMRI will, if anything, be underestimated compared with 3 T scanners.

The issue of disease threshold is perhaps the most contentious of issues within studies of this type. In order to calculate sensitivities and specificities, the disease entity that one is trying to rule in or rule out needs to be defined carefully. Our thresholds of risk (definitions 1 and 2) incorporated both volume and grade—the two most important determinants of risk in all cancers. Moreover, they were constructed around the two prevailing thresholds at the time: Stamey’s 0.5 cc and Epstein’s 0.2 cc, both volume-based definitions of risk [22], [29]. However, other studies have used different definitions, and there is no absolute consensus on which definition is the correct one. Indeed, we may need different definitions of risk over a person’s lifetime that would be contingent on a person’s life expectancy.

Given that mpMRI detects nearly all high-grade prostate cancers [1] and that these cancers are most strongly associated with prostate cancer–related death [30], it is possible that tumour visibility on mpMRI may confer useful prognostic information. However, this requires evaluation with long-term, mpMRI-correlated clinical trials. The suggestion that cancer not detected by mpMRI may be prognostically favourable compared with mpMRI-detected disease [16] is also reinforced by enrichment of aggressive molecular and microenvironmental features in mpMRI-visible tumours [17].

Disease volume and grade are strongly correlated with mpMRI visibility, but it is likely that there are other independent predictors of cancer conspicuity. In our analysis, we have shown that many of the tumours in PROMIS were of similar pathological grade. The majority of prostate cancers in PROMIS had an overall Gleason score of 3 + 4 (76% of mpMRI-detected tumours and 86% of mpMRI-undetected tumours), which suggests that Gleason grading alone may be inadequate to account for tumour conspicuity. Histopathologically, mpMRI inconspicuity may be related to a loose cellular and vascular arrangement of the tumour [13], [14], [15], thus more closely resembling background stromal tissue. This feature is shared with some histological prostate cancer subtypes (ductal and cribriform) that are also associated with reduced detection rates by mpMRI [31], [32]. To expand upon the post hoc analysis that is presented here, further in-depth radiological, histopathological, and biological investigation is underway to further elucidate the nature of mpMRI-inconspicuous disease.

## 5 Conclusions

On a per-patient basis, few significant prostate cancers remain undetected by mpMRI. The proportion of significant mpMRI-undetected cancers remains low, even at the upper limit of statistical estimates. Our post hoc analysis of the PROMIS cohort supports previous studies suggesting that prostate cancer undetected by mpMRI is lower in grade and size than the detected disease. These findings reinforce the key role that mpMRI plays in risk stratification of men with suspected prostate cancer. Further in-depth analysis of mpMRI-inconspicuous prostate cancer is currently being undertaken to enrich our understanding of the nature of undetected disease.

  ***Author contributions:*** Joseph M. Norris had full access to all the data in the study and takes responsibility for the integrity of the data and the accuracy of the data analysis.

*Study concept and design:* All authors.

*Acquisition of data:* Frangou, Brown, Freeman, Norris.

*Analysis and interpretation of data:* Carmona Echeverria, Emberton, Norris.

*Drafting of the manuscript:* Emberton, Norris.

*Critical revision of the manuscript for important intellectual content:* All authors.

*Statistical analysis:* Carmona Echeverria, Norris.

*Obtaining funding:* Norris.

*Administrative, technical, or material support:* None.

*Supervision:* Whitaker, Emberton.

*Other:* None.

  ***Financial disclosures:*** Joseph M. Norris certifies that all conflicts of interest, including specific financial interests and relationships and affiliations relevant to subject matter or materials discussed in the manuscript (eg, employment/affiliation, grants or funding, consultancies, honoraria, stock ownership or options, expert testimony, royalties, or patents filed, received, or pending) are the following: Norris and Stavrinides receive funding from the MRC. Carmona Echeverria receives funding from Prostate Cancer UK. Kirkham, Freeman and Emberton have shares in Nuada Medical Ltd. Hindley has stock or share interest with Nuada, is Clinical Director for the Prostate Care Division, and has also received funding from Sonacare for teaching and training. Punwani has sessional funding from UCLH BRC. Ahmed's research is supported by core funding from the United Kingdom's National Institute of Health Research (NIHR) Imperial Biomedical Research Centre. Ahmed currently receives funding from the Wellcome Trust, Prostate Cancer UK, The Urology Foundation, BMA Foundation, Imperial Healthcare Charity, Sonacare Inc., Trod Medical, and Sophiris Biocorp for trials in prostate cancer. Travel allowance was previously provided from Sonacare. Ahmed is a paid medical consultant for Sophiris Biocorp and Sonacare Inc. Ahmed is a proctor for Boston Scientific for Rezum and cryotherapy. Emberton receives funding from NIHR-i4i, MRC, Sonacare Inc., Trod Medical, Cancer Vaccine Institute, and Sophiris Biocorp for trials in prostate cancer. Emberton is a medical consultant to Sonacare Inc., Sophiris Biocorp, Steba Biotech, GSK, Exact Imaging and Profound Medical. Travel allowance was previously provided from Sanofi Aventis, Astellas, GSK, and Sonacare. Ahmed and Emberton are proctors for high-intensity focused ultrasound (HIFU) with Sonacare Inc. and paid for training other surgeons in this procedure. The other authors declare no competing interests.

  ***Funding/Support and role of the sponsor:*** PROMIS was funded by the UK Government Department of Health, National Institute of Health Research—Health Technology Assessment Programme (Project number 09/22/67). PROMIS was also supported and partly funded by UCLH/UCL Biomedical Research Centre and The Royal Marsden and Institute for Cancer Research Biomedical Research Centre. PROMIS was coordinated by the Medical Research Council Clinical Trials Unit (MRC CTU) at University College London (UCL). PROMIS was sponsored by UCL. The post hoc analysis presented here was funded by the MRC through an MRC Clinical Research Training Fellowship awarded to Joseph M. Norris (Grant Reference: MR/S00680X/1).

  ***Acknowledgements:*** The authors would like to thank Dr. Rhys Ball and Dr. Karen Scott for their kind assistance with pathological re-review of selected TRUS-biopsy slides.

## CRediT authorship contribution statement

**Joseph M. Norris:** Conceptualization, Methodology, Formal analysis, Investigation, Resources, Data curation, Writing - original draft, Writing - review & editing, Funding acquisition. **Lina M. Carmona Echeverria:** Formal analysis, Writing - review & editing. **Simon R.J. Bott:** Investigation, Writing - review & editing. **Louise C. Brown:** Software, Formal analysis, Resources, Data curation, Writing - review & editing. **Nick Burns-Cox:** Investigation, Writing - review & editing. **Tim Dudderidge:** Investigation, Writing - review & editing. **Ahmed El-Shater Bosaily:** Investigation, Resources, Data curation, Writing - review & editing. **Eleni Frangou:** Software, Resources, Data curation, Writing - review & editing. **Alex Freeman:** Conceptualization, Methodology, Investigation, Writing - review & editing, Supervision. **Maneesh Ghei:** Investigation, Writing - review & editing. **Alastair Henderson:** Investigation, Writing - review & editing. **Richard G. Hindley:** Investigation, Writing - review & editing. **Richard S. Kaplan:** Conceptualization, Methodology, Resources, Data curation, Writing - review & editing. **Alex Kirkham:** Conceptualization, Methodology, Investigation, Writing - review & editing. **Robert Oldroyd:** Conceptualization, Methodology, Writing - review & editing. **Chris Parker:** Conceptualization, Methodology, Writing - review & editing. **Raj Persad:** Investigation, Writing - review & editing. **Shonit Punwani:** Conceptualization, Methodology, Investigation, Writing - review & editing. **Derek J. Rosario:** Investigation, Writing - review & editing. **Iqbal S. Shergill:** Investigation, Writing - review & editing. **Vasilis Stavrinides:** Formal analysis, Data curation, Writing - review & editing. **Mathias Winkler:** Investigation, Writing - review & editing. **Hayley C. Whitaker:** Writing - original draft, Writing - review & editing, Supervision. **Hashim U. Ahmed:** Conceptualization, Methodology, Investigation, Writing - review & editing, Supervision, Funding acquisition. **Mark Emberton:** Conceptualization, Methodology, Investigation, Writing - original draft, Writing - review & editing, Supervision, Funding acquisition.
