# Supplementary material for: What Type of Prostate Cancer Is Systematically Overlooked by Multiparametric Magnetic Resonance Imaging? An Analysis from the PROMIS Cohort
Source: Eur Urol. 2020 Aug;78(2):163–70. doi: 10.1016/j.eururo.2020.04.029 (PMC7397509; doi:10.1016/j.eururo.2020.04.029)
Supplement: Supplementary file 1 [file mmc1.docx]

**Appendix A. Supplementary data**

**Supplementary Fig. 1 – Modified Barzell zone maps for each man with clinically significant mpMRI-invisible prostate cancer in PROMIS. Gl = Gleason; mm = millimetre.**

**Supplementary Fig. 2 – Scatter plot of PSA densities for both mpMRI-visible and mpMRI-invisible disease.**


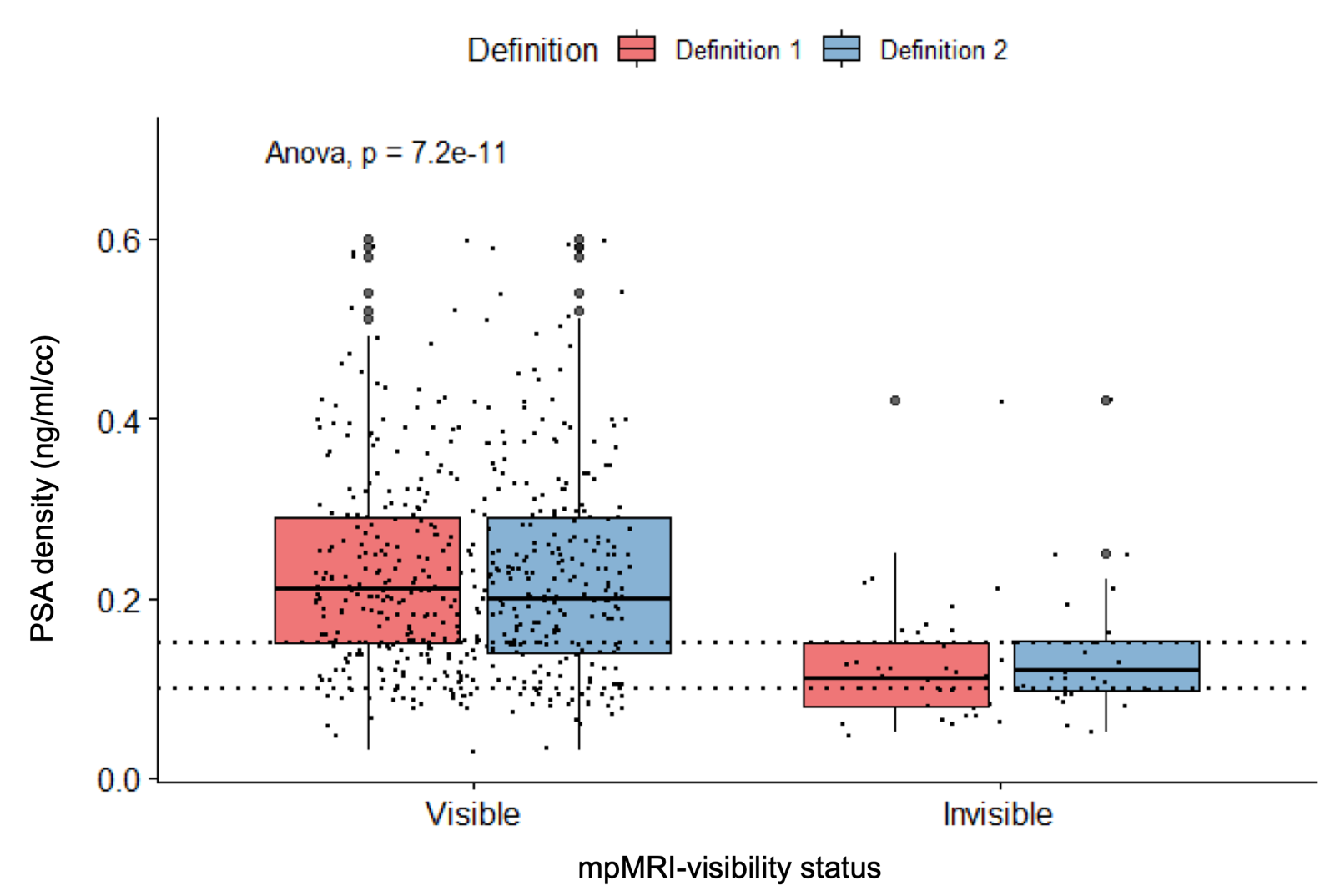


| **Pt** | **Age** | **PSA** | **PSAD** | **MRI score** | **Prost vol** | **Overall Gl (TPM)** | **Max Gl (TPM)** | **MCCL (TPM, mm)** | **Overall Gl (TRUS)** | **Max Gl (TRUS)** | **MCCL (TRUS, mm)** |
| --- | --- | --- | --- | --- | --- | --- | --- | --- | --- | --- | --- |
| 1 | 66 | 4.0 | 0.12 | 1 | 33 | 3+4 | 3+4 | 12 | 3+3 | 3+3 | 2 |
| 2 | 63 | 5.0 | 0.08 | 2 | 66 | 3+4 | 3+4 | 6 | 3+3 | 3+3 | 7 |
| 3 | 68 | 5.5 | 0.12 | 2 | 46 | 3+4 | 3+4 | 6 | 4+3 | 4+3 | 4 |
| 4 | 68 | 4.8 | 0.05 | 2 | 89 | 3+4 | 3+4 | 7 | 3+3 | 3+3 | 2 |
| 5 | 63 | 12.7 | 0.42 | 2 | 30 | 3+4 | 3+4 | 12 | 3+4 | 3+4 | 11 |
| 6 | 66 | 4.8 | 0.15 | 2 | 33 | 3+3 | 3+3 | 8 | - | - | - |
| 7 | 57 | 3.6 | 0.10 | 2 | 35 | 3+4 | 3+4 | 12 | 3+3 | 3+3 | 1 |
| 8 | 73 | 4.7 | 0.11 | 2 | 41 | 3+4 | 3+4 | 8 | - | - | - |
| 9 | 59 | 1.3 | 0.06 | 2 | 21 | 3+4 | 3+4 | 6 | - | - | - |
| 10 | 64 | 7.3 | 0.13 | 2 | 55 | 3+4 | 3+4 | 6 | 3+3 | 3+3 | 2 |
| 11 | 73 | 6.8 | 0.06 | 2 | 114 | 3+4 | 3+4 | 10 | 3+3 | 3+3 | 3 |
| 12 | 67 | 8.3 | 0.16 | 2 | 53 | 3+4 | 3+4 | 6 | 3+3 | 3+3 | 2 |
| 13 | 54 | 4.2 | 0.08 | 2 | 50 | 3+4 | 3+4 | 12 | 3+4 | 3+4 | 12 |
| 14 | 67 | 5.7 | 0.25 | 2 | 23 | 3+4 | 3+4 | 6 | 3+4 | 3+4 | 10 |
| 15 | 75 | 6.3 | 0.09 | 2 | 70 | 3+4 | 3+4 | 9 | 3+4 | 3+4 | 10 |
| 16 | 72 | 5.1 | 0.22 | 2 | 23 | 3+4 | 3+4 | 8 | 3+4 | 3+4 | 4 |
| 17 | 64 | 6.8 | 0.10 | 2 | 65 | 3+4 | 3+4 | 6 | 3+3 | 3+3 | 3 |
| 18 | 57 | 7.6 | 0.11 | 2 | 69 | 3+4 | 3+4 | 3 | 3+4 | 3+4 | 6 |
| 19 | 61 | 6.3 | 0.11 | 2 | 58.5 | 3+3 | 3+3 | 4 | 3+3 | 3+3 | 2 |
| 20 | 53 | 2.8 | 0.12 | 2 | 24 | 3+4 | 3+4 | 4 | 3+3 | 3+3 | 5 |
| 21 | 74 | 5.1 | 0.12 | 1 | 43 | 3+4 | 3+4 | 3 | 3+3 | 3+3 | 1 |
| 22 | 59 | 9.5 | 0.16 | 2 | 59 | 3+4 | 3+4 | 5 | 3+3 | 3+3 | 2 |
| 23 | 68 | 4.9 | 0.13 | 2 | 39 | 3+3 | 3+3 | 4 | 3+3 | 3+3 | 1 |
| 24 | 63 | 5.5 | 0.10 | 2 | 54 | 3+4 | 3+4 | 4 | - | - | - |
| 25 | 66 | 7.1 | 0.09 | 2 | 83 | 3+3 | 3+3 | 4 | - | - | - |
| 26 | 67 | 4.1 | 0.06 | 2 | 70 | 3+4 | 3+4 | 4 | - | - | - |
| 27 | 51 | 7.0 | 0.21 | 2 | 34 | 3+4 | 4+3 | 5 | 3+3 | 3+3 | 4 |
| 28 | 52 | 6.5 | 0.12 | 2 | 53 | 3+4 | 3+4 | 4 | 3+3 | 3+3 | 5 |
| 29 | 58 | 4.4 | 0.11 | 2 | 41 | 3+4 | 3+4 | 1 | - | - | - |
| 30 | 53 | 3.9 | 0.14 | 2 | 27 | 3+4 | 3+4 | 5 | - | - | - |
| 31 | 56 | 10 | 0.17 | 2 | 60 | 3+4 | 3+4 | 2 | - | - | - |
| 32 | 57 | 5.4 | 0.07 | 2 | 75 | 3+4 | 3+4 | 2 | 3+3 | 3+3 | 8 |
| 33 | 67 | 10.6 | 0.15 | 2 | 71 | 3+4 | 3+4 | 5 | 3+4* | 3+4* | 1.5 |
| 34 | 51 | 4.4 | 0.11 | 2 | 40 | 3+4 | 4+3 | 2 | - | - | - |
| 35 | 63 | 6.3 | 0.19 | 2 | 33 | 3+3 | 3+3 | 4 | - | - | - |
| 36 | 65 | 5.4 | 0.07 | 2 | 79 | 3+4 | 3+4 | 5 | - | - | - |
| 37 | 60 | 4.9 | 0.11 | 2 | 44 | 3+4 | 3+4 | 5 | - | - | - |
| 38 | 62 | 6.7 | 0.13 | 2 | 51 | 3+4 | 3+4 | 3 | - | - | - |
| 39 | 72 | 11.4 | 0.19 | 2 | 59 | 3+4 | 3+4 | 3 | 3+3 | 3+3 | 2 |
| 40 | 67 | 6.0 | 0.10 | 1 | 63 | 3+4 | 3+4 | 5 | - | - | - |
| 41 | 66 | 9.4 | 0.15 | 1 | 63 | 3+3 | 3+3 | 5 | - | - | - |
| 42 | 64 | 5.0 | 0.16 | 1 | 32 | 3+4 | 3+4 | 5 | 3+3 | 3+3 | 3 |
| 43 | 65 | 7.5 | 0.21 | 2 | 35 | 3+4 | 3+4 | 4 | 3+3 | 3+3 | 3 |
| 44 | 70 | 7.1 | 0.08 | 2 | 90 | 3+4 | 3+4 | 4 | 3+3 | 3+3 | 3 |
| Gl = Gleason score; max = maximum; MCCL = maximum cancer core length; mm = millimetre; MRI = magnetic resonance imaging; prost = prostate pt = patient; PSA = prostate specific antigen; PSAD = PSA density; TPM = template mapping biopsy; TRUS = transrectal ultrasound-guided biopsy; vol = volume; yr = year. * *Confirmed on whole-mount radical prostatectomy specimen.* | | | | | | | | | | | |

**Supplementary Table 1 – Characteristics of clinically significant prostate cancer not detected by mpMRI in PROMIS according to definition 1 (patients 1-17) and definition 2 (patients 1-44).**

**Supplementary Table 2 – Detection of clinically significant prostate cancer for each diagnostic modality for men with mpMRI-invisible disease according to definition 2, in PROMIS. Red = undetected disease; green = detected disease. mpMRI = multiparametric magnetic resonance imaging; TPM = template mapping biopsy; TRUS = transrectal ultrasound-guided biopsy.**

| **Patient** | **mpMRI** | **TPM** | **TRUS** |
| --- | --- | --- | --- |
| **1** |  |  |  |
| **2** |  |  |  |
| **3** |  |  |  |
| **4** |  |  |  |
| **5** |  |  |  |
| **6** |  |  |  |
| **7** |  |  |  |
| **8** |  |  |  |
| **9** |  |  |  |
| **10** |  |  |  |
| **11** |  |  |  |
| **12** |  |  |  |
| **13** |  |  |  |
| **14** |  |  |  |
| **15** |  |  |  |
| **16** |  |  |  |
| **17** |  |  |  |
| **18** |  |  |  |
| **19** |  |  |  |
| **20** |  |  |  |
| **21** |  |  |  |
| **22** |  |  |  |
| **23** |  |  |  |
| **24** |  |  |  |
| **25** |  |  |  |
| **26** |  |  |  |
| **27** |  |  |  |
| **28** |  |  |  |
| **29** |  |  |  |
| **30** |  |  |  |
| **31** |  |  |  |
| **32** |  |  |  |
| **33** |  |  |  |
| **34** |  |  |  |
| **35** |  |  |  |
| **36** |  |  |  |
| **37** |  |  |  |
| **38** |  |  |  |
| **39** |  |  |  |
| **40** |  |  |  |
| **41** |  |  |  |
| **42** |  |  |  |
| **43** |  |  |  |
| **44** |  |  |  |
